# Long-Term Sports Practice and Atrial Fibrillation: An Updated Review of a Complex Relationship

**DOI:** 10.3390/jcdd10050218

**Published:** 2023-05-16

**Authors:** Mattia Petrungaro, Liuba Fusco, Elena Cavarretta, Antonio Scarà, Alessio Borrelli, Silvio Romano, Renata Petroni, Flavio D’Ascenzi, Luigi Sciarra

**Affiliations:** 1 Unit of Electrophysiology, Belcolle Hospital, 01100 Viterbo, Italy; 2 Cardiology Department, University of Rome Sapienza, 00100 Rome, Italy; 3 Cardiology Unit, University Hospital of Northamptonshire, Northampton NN1 5BD, UK; 4 Department of Medical-Surgical Sciences and Biotechnologies, Sapienza University of Rome, 04100 Latina, Italy; 5 Mediterranea Cardiocentro, 80133 Naples, Italy; 6 Unit of Cardiology and Electrophysiology, San Carlo di Nancy Hospital, 00100 Rome, Italy; 7 Department of Life, Health and Environmental Sciences, University of L’Aquila, 67100 L’Aquila, Italy; 8 Department of Medicine, Di Lorenzo Clinic, 67052 Avezzano, Italy; 9 Department of Medical Biotechnologies, Division of Cardiology, University of Siena, 53100 Siena, Italy

**Keywords:** atrial fibrillation, arrhythmia, physical activity, sport, athletes

## Abstract

Atrial fibrillation (AF) is the most common sustained arrhythmia in clinical practice, and it is an enormous burden worldwide because of its high morbidity, disability and mortality. It is generally acknowledged that physical activity (PA) is strongly associated with a significant reduction in the risk of cardiovascular (CV) disease and all-cause mortality. Moreover, it has been observed that moderate and regular physical activity has the potential to reduce the risk of AF, in addition to improving overall well-being. Nevertheless, some studies have associated intense physical activity with an increased risk of AF. This paper aims to review the main related literature to investigate the association between PA and AF incidence and draw pathophysiological and epidemiological conclusions.

## 1. Introduction

Atrial fibrillation (AF) is the most common sustained arrhythmia in clinical practice, and it is an enormous burden worldwide because of its high morbidity, disability and mortality [1,2]. The prevalence of AF among the adult population is between 2% and 4%, with an increase in mortality between 1.5 and 3.5 times, and it has been associated with cardiovascular (CV) diseases such as heart failure, myocardial infarction, cerebral and ischemic events and all-cause mortality [3,4]. Its prevalence increases with age; indeed, more than 95% of AFs occur in those older than 60. AF is usually associated with cardiovascular and non-cardiovascular diseases [5]. However, this arrhythmia does not spare even young subjects with structurally normal hearts; this is the case of so-called lone AF [6].

It is generally recognized that physical activity (PA) is linked to a significant decrease in CV risk and all-cause mortality [7,8]. Moreover, it has been reported that moderate and regular physical activity has the potential to reduce the risk of AF [9,10].

Moderate and regular PA is considered a cornerstone in preventing AF by modifying many of its predisposing factors, which largely coincide with the classical CV disease risk factors [1,9,10,11,12,13]. Moderate, regular and aerobic physical activity improves many CV risk factors simultaneously, as opposed to drugs that are usually specific for a single factor [7]. Moderate PA can also be prescribed for subjects suffering from AF as part of their treatment because it improves muscular strength, ventricular rate control, 6 min walking test performance and quality of life [14,15]. The importance of PA in preventing AF seems to be further confirmed by the statistical correlation between a sedentary lifestyle and arrhythmia occurrence [16,17].

Nevertheless, some studies have associated intense physical activity with an increased risk of AF [18,19], reporting a higher incidence of AF in elite athletes [20,21,22] and suggesting a U-shaped dose–response curve [23,24]. These findings have raised doubts about the real beneficial effects of sports practice in arrhythmia prevention.

From a theoretical point of view, structural, functional and electrophysiological modifications induced by regular exercise may represent a favorable substrate for AF occurrence, leaving some questions still open:

1. How can sports practice prevent cardiovascular disease and, at the same time, increase the risk of AF?
2. Can we identify subjects that have an increased risk of developing sports-related AF or at least sports at higher AF risk?
3. What is the pathway to proper PA in cases with AF?

This paper aims to review the main related literature to investigate the association between PA and AF incidence and draw pathophysiological and epidemiological conclusions, providing some answers to open issues.

## 2. Possible Mechanisms of Atrial Fibrillation in Athletes

The exact mechanism of how PA can contribute to AF remains largely unclear and speculative. It is widely accepted that the initiation and perpetuation of AF require a specific combination of a trigger, a suitable substrate and a modulator. Given such triggers amongst athletes, the possibility of these playing a role in the development of AF is real. Considering the extensive literature supporting the beneficial impact of PA on health, the paradox that exercise might determine cardiovascular diseases deserves thorough investigation.

### 2.1. Electrophysiological Mechanisms of Atrial Fibrillation

Atrial fibrillation is a multifactorial arrhythmia. Its electrophysiology is complex and, even nowadays, not completely understood. Moreover, underlying arrhythmia mechanisms may differ depending on clinical scenarios and single patients [25,26], which can be crucial when considering athletes as “healthy” subjects.

**Figure 1:**
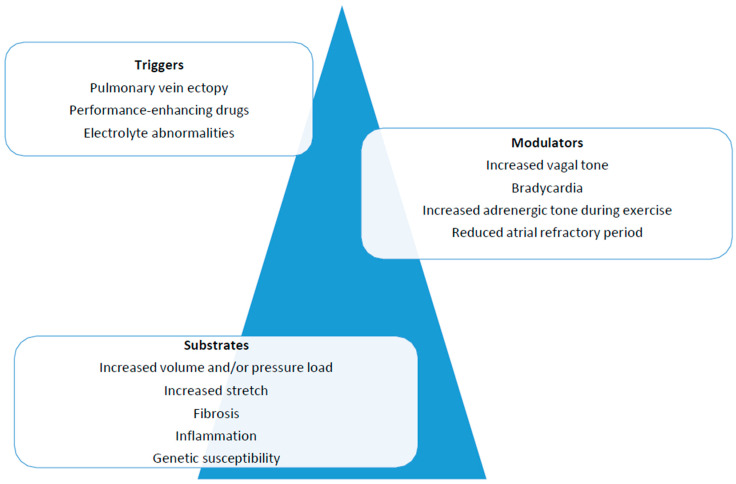
To support an arrhythmia, the presence of a trigger, a substrate and modulating factors is essential.

Research on this topic is not new and dates back to the early twentieth century, but significant advances have been made in the last 30 years, leading to our contemporary knowledge about this arrhythmia. In particular, the “Coumel triangle” marked a cornerstone in the history of arrhythmology (Figure 1) [27]. The triangle’s three corners represent the factors needed to support any type of arrhythmia: triggers, arrhythmogenic substrate and modulating factors. In this simple, although dated, model, AF still seems to fit adequately. These mechanisms have been proposed to play a crucial role in the genesis and sustainment of AF, and all factors of the triangle may occur in the same patient [28]. Generally speaking, the “trigger” is mainly identified as premature ectopic beats, often originating from inside the pulmonary veins’ ostia [29,30], but it can also be constituted by other supraventricular arrhythmias [26,31] that may degenerate into AF. The term “substrate” is a generic definition including all the structural, morphological and functional alterations that may favor arrhythmia maintenance. The “modulating factors” are mainly represented by sympathetic and vagal tone modifications, which in different clinical scenarios may favor the development of AF [17]. Understanding how these factors may be directly influenced by physical activity is intuitive.

### 2.2. Importance of the Specific Arrhythmia Triggers in Athletes

In the case of AF in a young athlete with a structurally normal heart, an underlying synchronized tachycardia inducing AF might be present. In the absence of any structural heart disease, AF is often triggered by other tachyarrhythmias. It is well known that some re-entrant tachycardias can induce atrial flutter or AF noted as “tachycardia-induced tachycardia” or “tachycardia-induced AF” [32,33].

In the general population, the occurrence of premature atrial contractions, particularly pulmonary vein ectopic beats, are the main trigger in most episodes of paroxysmal AF [29]. It has been postulated that endurance athletes present a higher burden of premature atrial beats than sedentary individuals [34,35]. Nevertheless, currently available data are insufficient to demonstrate a clear relationship between atrial premature beats and PA and whether such a little increase is enough to contribute to the AF burden in athletes significantly. In fact, Baldesberger et al. [36] and Elliot et al. [37] did not find an increased incidence of atrial ectopy in their study on former professional cyclists. Therefore, the exact role of atrial premature beats in athletes as prevalent triggers of AF remains unclear.

### 2.3. Arrhythmogenic Substrate Modification in Athletes

Intensive training leads to morphological and functional remodeling of the heart and cardiovascular system, resulting in the so-called “athlete’s heart” [38], which may play a role in the induction of AF. These CV adaptations are particularly evident when induced by endurance exercise to cope with the increase in cardiac output required during exercise and include biatrial dilatation [34], left ventricular hypertrophy [39] and enlargement of the right ventricle [40,41]. Usually, all anatomical changes disappear with detraining, but the exact amount of time required remains unclear [40]. Several factors related to exercise-related AF include atrial enlargement, an increased stretch of the atrium wall during activity and electrolyte disturbances (sodium, potassium, calcium and magnesium). It is possible to speculate that intense physical activity, combined with non- optimal electrolytic replenishment, can lead to an electrolyte imbalance that could trigger AF. However, the extent to which this aspect really contributes to the prevalence of AF in professional athletes is yet to be completely understood.

#### 2.3.1. Atrial Dilatation and Stretch

Atrial size and volume have been recognized as independent risk predictors for AF onset [42]. It is reported in the literature that a dilated left atrium may facilitate the establishment and maintenance of AF even in the absence of myocardial fibrosis [43]. Dilation of the left atrium is not uncommon in endurance athletes [34]. However, despite the training-induced dilatation, contrary to patients with structural heart disease, atrial enlargement in athletes is accompanied by a normal and even super-normal function as well as normal or even reduced atrial stiffness; this is true both in men and women practicing competitive sports as well as in children [44,45,46,47,48]. Recently, Cavigli et al. demonstrated that in master athletes running an ultra-marathon, acute exercise-induced biatrial dysfunction was not observed, as demonstrated by advanced echocardiography performed before and after the race [49]. However, this type of race is associated with a relevant electrolyte alteration that may favor the occurrence of AF irrespective of atrial dilatation. Therefore, training-induced atrial remodeling is currently considered a benign adaptation and one of the features of athlete’s heart and the current scientific evidence does not show a direct correlation between AF and sport-induced atrial remodeling.

#### 2.3.2. Fibrosis and Inflammation

Both inflammation and fibrosis are well-known risk factors for the development of AF in various clinical conditions [20,50,51]. Moreover, it is acknowledged that there is a direct relationship between AF and C-reactive protein [52]. That is why it is important to assess the influence of PA on systemic inflammation. On the one hand, studies show that intense exercise can generate a proinflammatory state with a transient increase in the neutrophil count and the release of proinflammatory cytokines [52,53,54,55]. On the other hand, sport has been shown to produce a systemic anti-inflammatory state [56,57]. During PA, parietal stretching of the left atrium may occur, which could cause inflammation and fibrosis. Indeed, some scientific work has highlighted the correlation between intense training, the presence of atrial interstitial fibrosis and increased risk of AF [58,59,60]. In active or former athletes, higher levels of profibrotic markers (such as galectin-3 and ST2) [52,53,54,55,56,57,58,59,60,61], circulating profibrotic microRNAs (miR-s), such as miR-21 [62], and collagen turnover peptide of collagen type I (PICP), carboxy-terminal telopeptide of collagen type I (CITP) and tissue inhibitor of matrix metalloproteinases type I (TIMP-1) [63] have been documented. Furthermore, these have been associated with incident or recurrent AF in clinical scenarios in the general population and in patients with structural heart disease [64,65]. In particular, some new profibrotic biomarkers such as circulating microRNAs (especially miR-1 and miR-26a) have been shown to play an important role in atrial remodeling [66]. Some studies have shown that vigorous endurance exercise can lead to an increase in circulating microRNA values [58,59,60,61,62,63,64,65,66,67,68]. In a recent study, in a transgenic goat characterized by overexpression of transforming growth factor β1 (TGF-β1)—which induces fibrosis—endurance exercise increased the incidence of spontaneous AF [69]. Finally, it was observed that an increase in AF inducibility occurs in the first two months of endurance exercise, concomitant with a decrease in circulating microRNA-21 and microRNA-29 [70,71].

However, these results do not allow us to state that there is a direct causal link between serum levels of circulating microRNA and AF in athletes, nor that increased atrial fibrosis may be directly responsible for AF in this population. Even if fascinating, more scientific evidence and further investigation are needed.

### 2.4. Possible Modulating Factor Modifications in Athletes

Autonomic tone can be significantly modified in athletes. Established cardiovascular adaptation to regular exercise is enhanced parasympathetic activity [72] which may generate sinus bradycardia at rest in well-trained individuals and AV conduction disturbances [73]. This is induced by increased sympathetic tone during exercise and a consequent downregulation at rest, which makes the vagal tone dominant [74,75,76].

This condition is reversible after detraining. Enhanced vagal tone and reduced sympathetic tone, characteristic of endurance athletes, have been associated with the development of AF, as well as in normal hearts [20,29,77]. Clinically, several features suggest that vagal tone can modulate AF, for example, when AF arises during sleep or after heavy meals. High levels of acetylcholine increase the dispersion of atrial repolarization and shorten the atrial effective refractory periods and the wavelength of atrial excitation wavefronts [20,59]. Notably, most AF relapses in athletes occur in vagally dominant situations while, conversely, adrenergic-mediated AF is less frequent in this population [22]. In a selected population of patients with paroxysmal vagal AF, vagal atrial denervation by radiofrequency ablation of right atrial ganglionated plexi was also attempted using an anatomic approach [78,79].

We should, however, consider that there are also other forms of AF: an adrenergic form and a combination of adrenergic and vagal forms. Increased sympathetic nervous system activity can theoretically trigger ectopic atrial activity and increase susceptibility to AF [29]. Undoubtedly, higher sympathetic tone is present in athletes during intense PA, but triggering AF during exercise is very rare [20]. From a purely theoretical point of view, modifications of autonomic tone in athletes could be involved in arrhythmogenesis in athletes. However, further studies are required to determine if isolated changes in the autonomic nervous system are sufficient to generate and maintain AF in such a population.

## 3. Association between AF and Sports Practice: A Summary of Literature

In recent decades, many papers have been published supporting a possible increased incidence of AF in sports practitioners.

Karjalainen et al. compared the prevalence of AF in middle-aged men former intense endurance sport athletes and men from the general population. The type and intensity of PA and the presence of cardiac arrhythmias were investigated with a questionnaire that patients completed one and eleven years after enrolment. A higher prevalence of lone AF in athletes (5.3%) than in men from the general population (0.9%) was reported [20].

A few years later, Mont et al. [22] concluded that continual vigorous exercise could be a risk factor for AF in men. They analyzed 1160 consecutive patients visiting the Outpatient Arrhythmia Clinic between October 1997 and March 1999, recording 70 young patients suffering from lone AF of which about 50% had been practicing endurance sports for a long time. In a follow-up study, the same scientific group compared the incidence of lone AF in a cohort of marathon runner men and sedentary subjects of the general population followed for 11.3 and 6.4 years, respectively. They recorded an annual incidence rate of lone AF about four times higher in athletes and concluded that chronic endurance sport practice is associated with a higher risk of AF in men [80,81].

Moreover, Furlanello et al. [21] came to similar conclusions; they analyzed the presence of AF in a population of 146 young elite athletes who suffered from palpitations, including previous Olympic and world champions, in a follow-up of 62 months. The prevalence of AF was 9%, and all the athletes with this arrhythmia were men.

More recently, in the Physicians’ Health Study [82], 16,921 men practicing vigorous exercise were studied in a 12-year follow-up. During this period, 1661 sportsmen developed AF. An increased incidence of AF was correlated to an increased level of PA; in particular, joggers who trained 7 days/week had a 20% higher risk of AF. Other evidence supporting the relationship between AF and sports practice is found in a meta-analysis by Abdulla J [10], who analyzed 655 athletes compared with 895 controls: an increased AF risk was shown in athletes engaged in long-term physical endurance activity. Another study [82] compared the prevalence of AF in swimmers over 60 years of age and in some patients admitted to internal medicine. The authors concluded that long-term competitive swimmers had a higher incidence of AF (26.5% vs. 7%), despite a lower prevalence of cardiovascular risk factors such as diabetes mellitus and hypertension.

Therefore, a global review of such papers, despite the heterogeneity of the studies, seems to show an increased AF risk in men practicing endurance sports, yielding the conclusion that sports practice could be harmful in some cases, raising important doubts in the scientific community.

Indeed, Marijon E. et al. [83] investigated mortality among 786 cyclists and participants in the Tour de France (between 1947 and 2012) by comparing them with the general male population and observed significantly lower mortality (40% less) in athletes.

In addition, a study [84] of 15,000 Olympic champions from 9 different countries shows a progressive increase in survival for Olympic champions compared to the general population, regardless of some indicators such as country, medal or type of sport. Furthermore, the same authors found that especially endurance sports athletes had an increase in survival.

However, the global analysis of the previous works seems to suggest a higher incidence of AF in various populations of sportsmen. Nevertheless, other evidence in the literature does not seem to confirm this theory.

Currently, the relationship between BP and AF, especially in females, appears fuzzy. In fact, a meta-analysis [85] included 95,526 women, comparing the incidence of AF in those who practiced sports versus those who had a sedentary lifestyle. Analysis of the results showed that those who practiced sports had a decreased cardiovascular risk and no increased incidence of AF. Moreover, in a recent prospective cohort study (Rotterdam Study), Albrecht M et al. [86] investigated the relationship between PA and AF.

The authors studied 7018 people aged 55 years and older, with a median follow-up of 12.3 years, and analyzed total physical activity differing between types of sports (walking, cycling, domestic work and gardening). They observed neither PA in general nor any of the included physical activity types to be associated with AF risk, concluding that physical activity is not a cause of AF in adults. However, while there is uncertainty about the benefits of extreme endurance sports, there is no doubt that mild-to-moderate physical activity is not linked to the risk of AF and indeed should be recommended. The Cardiovascular Health Study [12] followed 5446 adults aged 65 years or older (58% women) for more than 12 years and revealed that, in contrast to high-intensity exercise, light–moderate physical activity, particularly leisure time activity and walking, was associated with a lower incidence of AF. Finally, some studies have investigated whether female or male sex may be a predisposing, protective or neutral factor in the development of AF in athletes. A prospective study of more than 400,000 individuals (mean age 56 ± 8 years; females 52.4%; median follow-up 7 years) appears to show a different relationship between sport and AF in males and females [87]. While in men a high amount of vigorous PA was associated with an increased incidence of AF (12%), in women, in contrast, vigorous physical activity was associated with an 8–16% reduction in the incidence of AF. It thus appears that in women, vigorous physical activity is also associated with a lower risk of AF. Furthermore, in a recent and extensive meta-analysis of a single cohort with 2 million people, Kunutsor et al. [88] reported an increased risk of AF in men and a reduced risk in women, confirming that the regular sport–AF association is sex-linked.

## 4. European and Italian Guidelines

An important question is whether athletes with AF can be eligible for competitive sports. In Italy, since 1989, an expert committee, including five national scientific societies, has produced a list of recommendations (COCIS) for competitive sports eligibility and disqualification. The latest English version of these guidelines was published in 2021 [89]. A general difference between Italian and European guidelines reflects the peculiarity of Italian law: in Italy, every competitive athlete has to be judged as eligible by a sports medicine doctor, and this eligibility has to be renewed annually. On the other hand, the European guidelines [90] indicate good practice for people engaging in PA at various levels. In contrast, the COCIS guidelines refer specifically to competitive athletes in various sports, including those with high cardiovascular stress. For this reason, Italian guidelines tend to be more restrictive than European ones.

The COCIS guidelines consider either paroxysmal or persistent AF or permanent AF. If a paroxysmal or persistent AF is found in an apparently healthy young man, guidelines recommend performing echocardiography, exercise stress test, ECG Holter monitoring and, if necessary, transesophageal or endocavitary electrophysiological study. The endocavitary electrophysiological study may identify some synchronized triggers of the arrhythmia. Professional sport is permitted in the absence of heart disease, if a possible trigger has been identified and removed (hyperthyroidism, alcohol abuse, illicit drugs, etc.), if there is no cause–effect relationship between sports activity and the occurrence of AF, if arrhythmic episodes are short, asymptomatic and not frequent and in the absence of Wolff–Parkinson–White syndrome. In the presence of heart disease, eligibility depends on the underlying cardiovascular disease.

Regarding permanent AF, eligibility can only be granted for sports with low cardiovascular demand and in the absence of organic heart disease, hemodynamic impairment and/or symptoms during exercise and marked bradycardia or bradycardia-induced tachyarrhythmias. In addition, patients on anticoagulant therapy who participate in sports at high risk of trauma must be excluded from sports eligibility.

The problem of AF in athletes was also addressed in the 2020 ESC guidelines [90] on sports cardiology and exercise in patients with cardiovascular diseases. Similar to the COCIS guidelines, before recommending sports activity in subjects with recognized AF, it is always necessary to rule out an underlying structural heart disease or pre-excitation and to look for secondary causes of AF (drugs, dysthyroidism, alcohol). Sporting eligibility is possible in asymptomatic subjects with evidence of adequate exercise frequency control [91]. In addition, the ESC guidelines [90] also discuss the therapeutic approach for these patients: the pill-in-the-pocket approach using class I antiarrhythmic drugs is recommended in patients with paroxysmal AF with avoidance of sport until two half-life periods of the antiarrhythmic drug have elapsed [90]. An important role is given to catheter ablation, which should be considered if drug therapy fails or as first-line therapy if drug therapy is not desired (class I level B) [90].

As athletes have a high prevalence of bradycardia and sinus pauses, medical therapy is frequently contraindicated or poorly tolerated. Therefore, achieving adequate rate control can be difficult. Beta-blockers may not be tolerated and, in some sports, are considered doping drugs. Calcium-channel blockers and digitalis are usually not adequate when used alone. Pharmacological rhythm control strategy may be complex in athletes: class III antiarrhythmic drugs are often ineffective or contraindicated in young people [90]. Moreover, class I antiarrhythmics in monotherapy may alter the ventricular rates during AF or atrial flutter [90].

## 5. Discussion

When reviewing the scientific papers investigating the relationship between PA and AF, it is clear that this relationship is controversial. Indeed, while some articles emphasize the beneficial effect of PA in general and even suggest it as a protective factor for the development of AF, other studies report a clear association between the incidence of AF and sporting activity.

It is therefore necessary to clarify these contradictions. First of all, it is possible to identify whether certain confounding factors may have influenced the homogeneity of the data published in the literature. For example, it is possible that the higher incidence of AF in athletes observed in some scientific studies may simply have been caused by a selection bias. In fact, it is well known that athletes, even if asymptomatic, undergo medical examinations more frequently than non-athletes. In Italy, for example, athletes are legally obliged to undergo medical examinations annually in order to obtain sporting eligibility. However, whether this selection bias exists and how important it might be are fascinating questions on which there is a lack of prospective, controlled studies.

Another confounding factor that may erroneously increase the prevalence of AF in sportspeople is the easier symptomaticity that athletes may manifest. Indeed, although AF may occur asymptomatically at rest, especially in young individuals, asthenia and easy fatigability may develop during PA, prompting the patient to seek medical attention.

Another aspect to consider is the increase in sporting activity in master populations. In fact, it is known that the prevalence of AF increases with age [1] and the higher prevalence of master athletes could in part explain the elevated prevalence of AF in certain athlete populations.

Another misleading factor regarding the incidence of AF in athletes is the use of illicit substances. Indeed, many illicit substances are sympathetic nervous system stimulants and may trigger arrhythmias such as AF. Unfortunately, due to the obscure nature of doping, it is impossible to quantify the problem and come to a scientific conclusion, so this issue remains only speculative.

However, even considering the possible presence of confounding factors in the AF–PA relationship, it cannot be denied that some kinds of sport may generate a substrate that could facilitate the onset and maintenance of AF. Furthermore, as reported in this review, there are numerous scientific studies which seem to suggest that certain types of sport, particularly endurance sports, may facilitate the development of AF. Therefore, the AF–PA relationship is a complex one which seems to be influenced not only by the type of sport but also by its intensity and duration.

Some papers [23,24] report that the relationship between AF and sport may be a “U-shaped” relationship whereby both a sedentary lifestyle and strenuous, long-duration PA may prove to be risk factors for AF. A lower risk of AF has been reported in patients practicing sport of moderate intensity or duration, but not during vigorous exercise [16]. However, the upper safe limit for endurance PA beyond which sport switches from being a protective factor to a risk factor has not been defined. Indeed, while Elosua R et al. [91] reported that more than 1500 h of sport was associated with an increased risk of AF, Calvo Net et al. [92] indicated that the safe threshold was 2000 h in endurance sports. However, even if a defined “safe” limit is difficult to identify, we can generally conclude that “much is too much”, especially in predisposed individuals.

Even though many endurance athletes develop adaptations of the cardiovascular system, mainly the so-called athlete’s heart, most do not develop AF. This should lead one to consider other factors that are probably important in the genesis of AF in athletes, such as genetic predisposition. Indeed, typical cardiovascular risk factors that contribute to the development of AF in non-athletes (smoking; diabetes mellitus; hypertension; obesity; chronic bronchopneumopathy; and sleep apnea) cannot be attributed to the athlete population as these usually have a low prevalence in this cohort.

Some studies have identified several single nucleotide polymorphisms associated with an increased risk of developing AF, even in non-athletes [93,94]. Therefore, it is possible that in some athletes, the presence of a sport-induced substrate combined with the presence of certain genetic polymorphisms may amplify the risk of AF. Unfortunately, further studies are badly needed to better clarify this aspect, mainly for male athletes.

What, on the other hand, is known about the relationship between PA and AF in women? Although it is common for female athletes to be excluded from scientific studies, there is some evidence that not only is there no increased incidence of AF in endurance athletes, but that female sex is a protective factor against AF, even in female athletes. This is supported by large prospective studies [87] and a meta-analysis conducted on approximately 2 million athletes [88].

A recent study conducted on female amateur marathon runners investigated the remodeling of the cardiac chambers induced by endurance training, which might be different from that of male athletes [95]. The authors [95] found that immediately after a marathon there was a significant, but reversible, dilation of both the right atrium and the right ventricle, accompanied by a decrease in the contractility of the right ventricle. In contrast, the left heart chambers were less affected by acute remodeling: the size of the left ventricle had decreased after the race and no significant reduction in the size of the left atrium was observed. This is a very interesting study even if it does not investigate the possible long-term consequences of repeated strenuous training.

## 6. Conclusive Considerations

**Figure 2:**
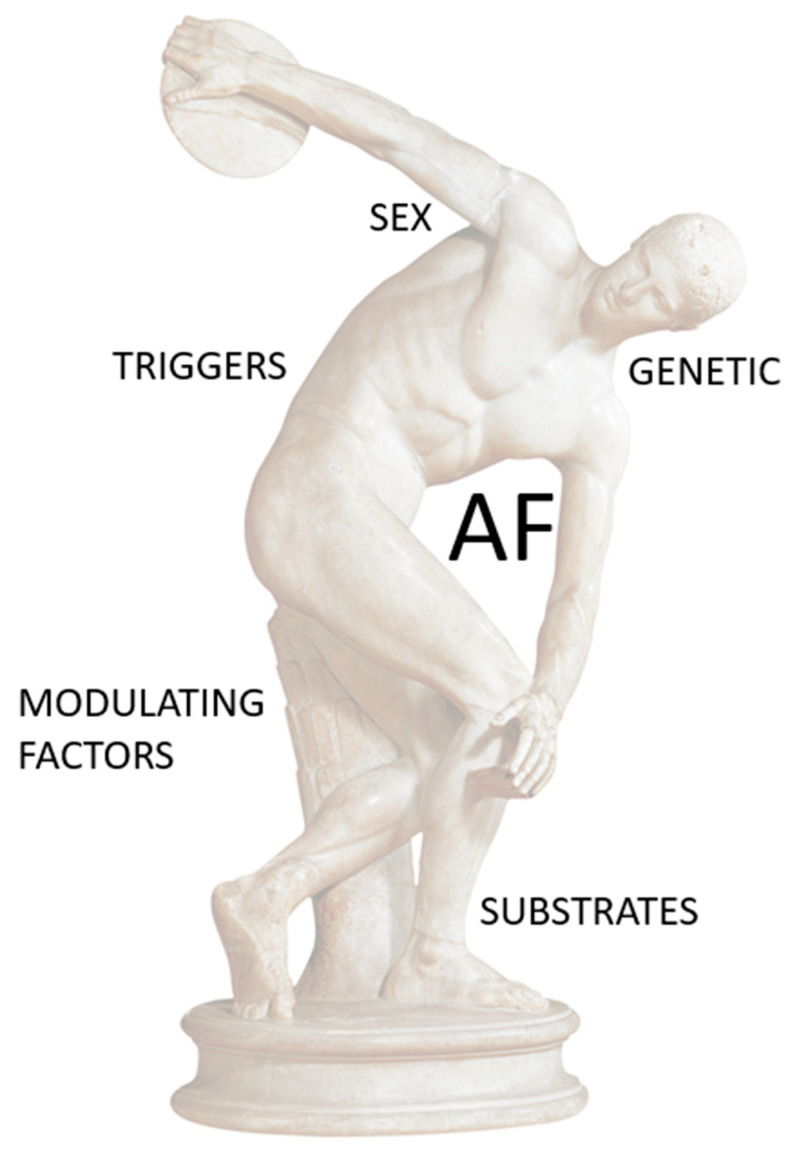
The AF–PA relationship is controversial and can be influenced by numerous components.

In conclusion, The AF–PA ratio is controversial and can be influenced by numerous components (Figure 2). However, current scientific evidence converges in confirming that regular exercise with mild to moderate intensity reduces the risk of AF, as opposed to a sedentary lifestyle that is a risk factor for the development of this arrhythmia. For these reasons, the prescription of PA should be encouraged in all patients, including those with AF. We believe that generating doubts about the beneficial effect of PA can be somewhat dangerous and is not scientifically justified.

However, while there is insufficient scientific evidence to state with certainty that some kinds of sport may be a risk factor for AF, it is reasonable to state that endurance sports practiced at high intensity may promote AF, particularly in men, master athletes and those with a genetic predisposition.

On the other hand, scientific data seem to confirm that in female athletes, physical activity does not lead to an increased incidence of AF, thus concluding that female sex is a protective factor against AF, possibly due to a different exercise-induced remodeling of the heart chambers.

Lastly, large, prospective clinical studies are strongly recommended to improve knowledge of the pathophysiological relationships between PA and AF, allowing early identification of at-risk athletes and which type of sport and what intensity may favor the development of AF.

## Data Availability

No new data were created or analyzed in this study. Data sharing is not applicable to this article.
